# The Quality of Methodological and Reporting in Network Meta-Analysis of Acupuncture and Moxibustion: A Cross-Sectional Survey

**DOI:** 10.1155/2021/2672173

**Published:** 2021-01-11

**Authors:** Ting Yuan, Jun Xiong, Xue Wang, Jun Yang, Yunfeng Jiang, Xiaohong Zhou, Kai Liao, Lingling Xu

**Affiliations:** ^1^Institute of Acupuncture, Moxibustion and Tuina, Jiangxi University of Traditional Chinese Medicine, Nanchang, Jiangxi, China; ^2^Department of Acupuncture and Moxibustion, The Affiliated Hospital of Jiangxi University of TCM, Nanchang, Jiangxi, China

## Abstract

**Background:**

Acupuncture had long been a primary treatment in the healthcare system of China. In recent years, there were more and more network meta-analyses (NMAs) in the field of acupuncture and moxibustion, but the quality evaluation of NMAs was rare.

**Objectives:**

The goal of this study was to evaluate the methodological and reporting quality of NMAs and summarize the effects of different treatments of acupuncture and moxibustion.

**Methods:**

PubMed, Embase, the Cochrane Library, China National Knowledge Infrastructure Database (CNKI), WanFang Database (WF), Chinese Scientific Journal Database (VIP), and Chinese Biomedical Literature Database (CBM) were searched from inception to January 2020 without any language restriction. In addition, the unpublished studies and the references of initially included literature were also retrieved manually. We included all relevant NMAs treated with acupuncture and moxibustion; other therapies such as traditional Chinese medicine and Western medicine may also be included, but at least three types fall under the category of acupuncture in each NMA. Outcome indicators were not limited. We selected AMSTAR2 and PRISMA-NMA to evaluate the methodological and reporting quality of eligible studies, respectively.

**Results:**

In total, 29 NMAs were included finally, including 12 Chinese references and 17 English references. All eligible studies were published from May 2013 to August 2019. The number of interventions was between 4 and 22. The number of clinical trials included ranged from 10 to 121, with a total of 1098 clinical trials. The NMAs were involved in up to 23 diseases, knee osteoarthritis and primary dysmenorrhea covered with 3 NMAs separately, others focusing on chronic functional constipation, lumbar disc herniation, chronic fatigue syndrome, and the like. The Jadad scale and RoB scale were used as the bias risk assessment tools. Among them, 7 articles adopted the Jadad scale, 22 articles adopted the RoB scale (1 article adopted both the Jadad scale and RoB scale), and only 1 article did not mention the risk assessment tool. The AMSTAR2 methodological evaluation showed that the highest score was 13.5 points and the lowest was 4, with an average of 8.64 and a median of 9.5. According to the quality criteria, only one of them was in high quality, twenty-four were in medium quality, and four were in low quality. The PRISMA-NMA reporting quality evaluation showed that the highest score was 29 points and the lowest was 13.5, with an average of 23.62 and a median of 24.5; severe flaws also existed in some items, especially in “Structured summary,” “Protocol and registration,” “Search,” “Data collection process,” “Data items,” “Additional analyses,” “Risk of bias across studies,” and “Results of additional analyses.”

**Conclusion:**

The number of NMAs in the field of acupuncture and moxibustion was still in the initial stage. Overall, their methodology and reports were of moderate quality. However, severe flaws also existed in some items. Because the eligible NMAs were limited, the conclusion needed further research to confirm its authenticity and reliability.

## 1. Introduction

Acupuncture has a history of more than 4,000 years and is now commonly used in medical care in China. As a traditional oriental therapy, it has been widely used globally [1]. With the development of evidence-based acupuncture and moxibustion, acupuncture and moxibustion is more and more popular in clinics because of its simple treatment, quick effect, and nontoxic side effects. However, there are many kinds of acupuncture and moxibustion intervention methods, and the indications are similar. Traditional meta-analysis can only achieve pairwise direct comparison of intervention measures, but it cannot carry out indirect comparison of intervention measures without a direct comparative study, and let alone comparative analysis of various intervention measures; so, it is difficult to solve this problem.

Network meta-analyses (NMAs) are still named multiple-treatments meta-analysis (MTM) or mixed treatment comparison meta-analyses (MTC). Meta-analysis is an analytical method that evaluates the relative efficiency of treatments and synthesizes evidence using a randomized controlled trial network diagram. This method is based on the combination of traditional direct/head-to-head comparison and indirect comparison for meta-analysis, which can simultaneously compare the efficacy of three or more interventions. It was developed on the basis of classical meta-analysis, which resulted in a consistent and complete set of comparisons based on all available evidence from existing research studies [2–5]. The NMA provides evidence for clinical drug selection by quantifying different interventions to treat the same disease and ranking the benefits [6]. As an emerging evidence synthesis tool, NMAs are becoming more and more popular, which can make more decisions or choices than classic meta-analysis [7–10].

In recent years, NMA has made some progress in the field of acupuncture and moxibustion, and the number of publication of NMAs related to acupuncture and moxibustion is on the rise, but its quantity is still small, and its quality still lacks systematic evaluation. This study through retrieving NMAs of acupuncture and moxibustion published a comprehensive understanding of the present situation, and apply AMSTAR2 (a measurement tool to assess the methodological quality of systematic reviews) and PRISMA-NMA (PRISMA extension statement for reporting of systematic reviews incorporating network meta-analyses of health care interventions: checklist and explanations) to assess the methodological and reporting quality of the published NMAs in acupuncture and moxibustion field systematically, in order to offer reference to improve the quality of acupuncture and moxibustion in NMA.

## 2. Materials and Methods

### 2.1. Information Sources

PubMed, Embase, the Cochrane Library, China National Knowledge Infrastructure Database (CNKI), WanFang Database (WF), Chinese Scientific Journal Database (VIP), and Chinese Biomedical Literature Database (CBM) were searched from inception to January 2020 without any language restriction. In addition, the unpublished studies and the references of initially included literature were also retrieved manually. The comprehensive search strategy for PubMed is presented in Table 1. The retrieval of other electronic databases was similar to PubMed, which adopts the combination of subject words and keywords.

### 2.2. Eligible Criteria

We included all relevant NMAs treated with acupuncture and moxibustion; other therapies such as traditional Chinese medicine and Western medicine may also be included, but at least three types fall under the category of acupuncture in each NMA. Outcome indicators were not limited, while language limited in Chinese and English.

### 2.3. Exclusion Criteria

(1) Duplicate detection and republished literature. (2) Theoretical research. (3) Reviews, conference papers, and abstracts. (4) Incomplete data of the results. (5) Acupuncture interventions <3. (6) Non-Chinese and English literature.

### 2.4. Study Selection and Data Extraction

The search was conducted by NoteExpress 5.3.0 literature management software. NoteExpress 5.3.0 automatic duplicate check function was used and combined with manual duplicate check to eliminate the duplicate research. By reading the title and abstract, we excluded studies that obviously do not meet the inclusion criteria; downloaded and read the full text of the remaining studies to judge; and for research with incomplete data report, tried to contact the author and gain the complete data. The three evaluators (TY, XW, and JY) screened and extracted the literatures back to back independently according to the inclusion and exclusion criteria, and cross-checked the results. The included details were as follows: author information, year of publication, sample size, disease, type of study, diagnostic criteria, number of original study, description of interventions, number of interventions, comparators, outcome measures, and risk assessment tools for bias and adverse reactions. When there was any disagreement, it was resolved by the fourth researcher (JX).

### 2.5. Methodological and Reporting Quality Assessment Tools

Three independent researchers (TY, XW, and JY) evaluated the methodological and reporting quality back to back. The AMSTAR2 tool contained 16 aspects [11]. For each aspect, when the answer was “yes,” the score was 1, and when the answer was “no,” “cannot answer,” or “not applicable,” the score was 0. The total score of an NMA was calculated by counting the number of “yes” in 16 items on a scale of 0–16. A score of 12–16 was rated as “high quality,” a score of 7–11 was rated as “medium quality,” and a score of 0–6 was rated as “low quality.” The PRISMA-NMA contained 32 items [12]. For each item, a score of “1” means full compliance, “0.5” means partial compliance, and “0” means noncompliance [13, 14]. The total PRISMA-NMA score of an NMA was calculated by accumulating the scores of each item, with a range of 0–32. A score of 26–32 was rated as “high quality,” a score of 20–25.5 as rated as “medium quality,” and a score of 0–19.5 as rated as “low quality.” When there was any disagreement, it was resolved by the fourth researcher (JX).

### 2.6. Data Analysis

We analyzed the characteristics of included studies through descriptive statistical methods. All the data used were counted in the Excel 2007 spreadsheet. We described the dichotomous data in terms of number and percentage and the continuous variables in terms of median with interquartile range (IQR). And we calculated the number of papers per item, its percentage, and 95% confidence intervals. We summarized the scores according to the quality evaluation tool. AMSTAR2 and PRISMA-NMA scored 16 and 32, respectively. Finally, we calculated the total score through adding a list of each component.

## 3. Results

### 3.1. Search Results

**Figure 1:**
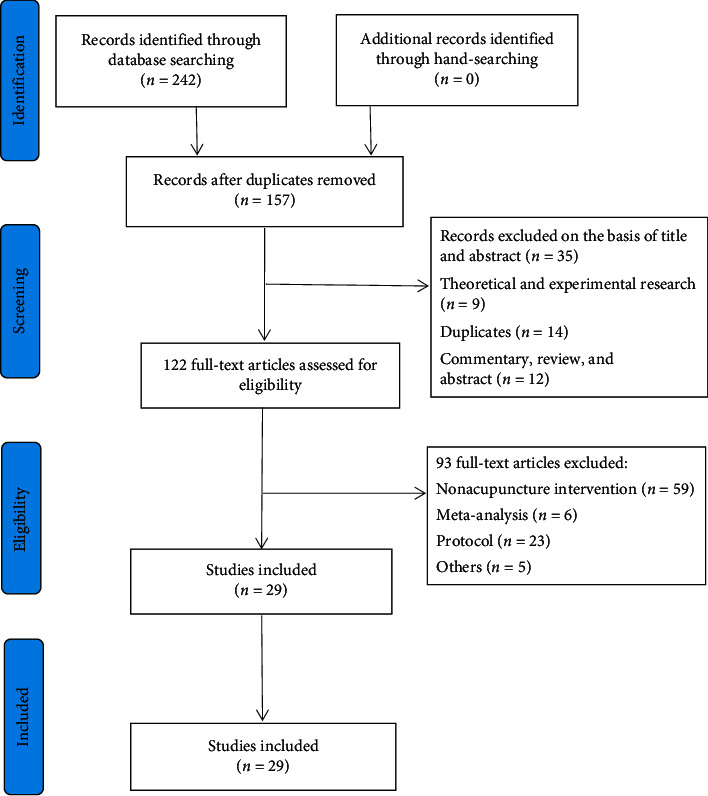
Flow diagram of the study.

242 related references through searching seven electronic databases and manual searches were retrieved. After reading the title and abstract, a total of 29 studies were included [15–43]. The literature screening process is shown in Figure 1.

### 3.2. Study Characteristics

The characteristics of all included NMAs were documented. After screening, 29 NMAs finally met the inclusion criteria, including 12 Chinese references (including 1 master's thesis) and 17 English references. All eligible studies were published from May 2013 to August 2019. The study contained 4–22 types of treatments and 10–121 RCTs for 1098 RCTs in total. The NMAs were involved in up to 23 diseases, knee osteoarthritis and primary dysmenorrhea covered with 3 NMAs separately, others focusing on chronic functional constipation, lumbar disc herniation, chronic fatigue syndrome, and the like. The Jadad scale and RoB scale were used as the bias risk assessment tools. Among them, 7 articles adopted the Jadad scale, 22 articles adopted the RoB scale (1 article adopted both the Jadad scale and RoB scale), and only 1 article did not mention the risk assessment tool. The characteristics of the eligible studies are presented in Table 2.

### 3.3. Methodological Quality Assessment

**Figure 2:**
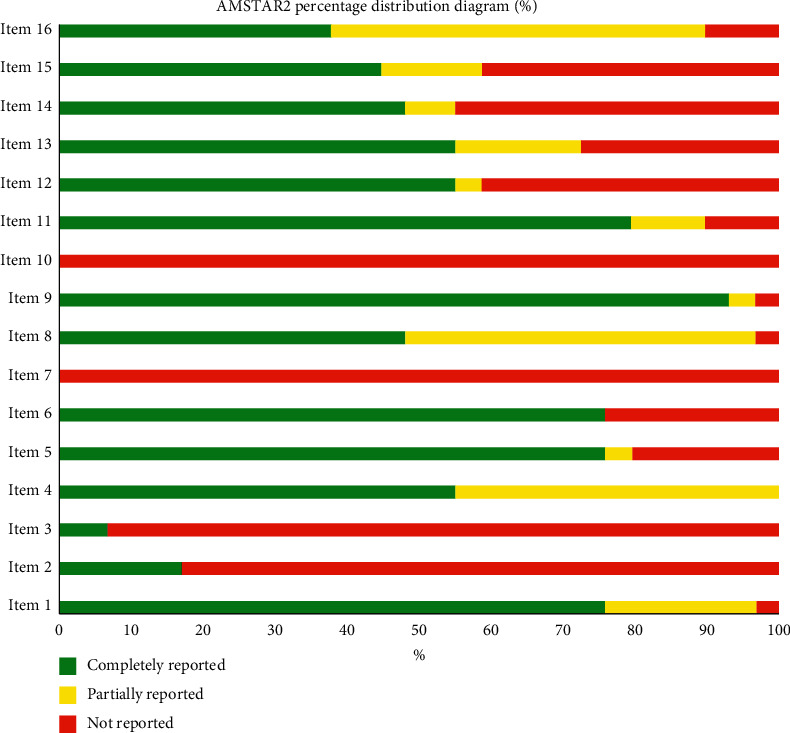
AMSTAR2 percentage distribution diagram (%).

According to the AMSTAR2 checklist, the median score and IQR of eligible NMAs was 9.5 (6.5–10.75), and the details are presented in Tables 3 and 4. The item with the best degree of compliance was “comprehensive literature search” (100%) (Table 4). Longitudinal analysis, a good degree of compliance was with item 9 (93.1%), item 11 (79.31%), and items 1, 5, and 6 (75.86%). However, a poor degree of compliance was with item 2 (17.24%) and item 3 (6.9%), and the worst degree of compliance was with item 7 (0%) and item 10 (0%).The details are presented in Tables 3 and 4 and Figure 2.

### 3.4. Reporting Quality Assessment

According to the PRISMA-NMA checklist, the median score and IQR of eligible NMAs was 24.5 (20.5–26.5).The item with the best degree of compliance was item 1 “Title” (100%). Longitudinal analysis, a good degree of compliance was with item 4 (96.55%), item 12 (96.55%), item 25 (96.55%), item 18 (93.10%), item 26 (93.10%), item 3 (89.65%), item 19 (89.65%), and item 24 (86.21%). However, a poor degree of compliance was with item 23 (27.59%), item 2 (17.24%), item 5 (17.24%), item 10 (17.24%), and item 11 (10.34%), and the worst degree of compliance was with item S4 (3.45%). There are significant problems in the reporting methods and results sections, such as underreporting or selective reporting. Three studies [29, 39, 42] (10.34%) reported evaluating the risk of bias within individual studies in the methods section (item 12), but did not really evaluate it in the results section (item 19). Horizontal analysis, there were four NMAs (13.79%) that scored less than 20 points, with a minimum score of 13.5. The details are presented in Tables 5 and 6.

## 4. Discussion

### 4.1. Summary of Main Findings

The goal of this cross-sectional survey was to evaluate the methodological and reporting quality of NMAs and summarize the effects of different treatments of acupuncture and moxibustion (Table 6). A total of 29 acupuncture NMAs were included in this study. From the perspective of the number and publication time, the development of NMA in acupuncture and moxibustion was still in the initial stage, with a small number, but it had shown a trend of gradual growth. In terms of disease, more than half were chronic pain. In terms of intervention measures, more than half were acupuncture combined therapy. It was not difficult to find that NMAs of acupuncture and moxibustion was still limited to several diseases, and there were still large gaps in many aspects.

The methodological quality of NMAs was important, so we evaluated the methodological quality of NMAs in acupuncture and moxibustion according to AMSTAR2 tool. The results showed some methodological deficiencies, particularly with regard to item 2, item 3, item 7, and item 10. The highest NMA score for each item was 13.5, the lowest was 2.5, and the median and IQR was 9.5 (6.5–10.75), indicating average methodological quality.

The quality evaluation of the report showed that the quality of acupuncture NMAs was generally acceptable, indicating that the NMA researchers had a high level of evidence-based medical knowledge and scientific research literacy. Some items need to be improved, particularly with regard to the structured summary (item 2), protocol and registration (item 5), search (item 8), data collection process (item 10), data items (item 11), additional analyses (item 16), risk of bias across studies (Results section) (item 22), and results of additional analyses (item 23). From the perspective of a single NMA, the highest score was 29, the lowest was only 13.5, and the median and IQR was 9.5 (6.5–10.75), showing that the quality of reports included in the study was of average quality. PRISMA-NMA checklist was helpful to improve the reporting quality of acupuncture NMAs. Therefore, it was necessary to improve the comprehensiveness and standardization of the report.

### 4.2. Strengths and Limitations

First, this was the first study that evaluated the methodological and reporting quality of NMAs comprehensively, which complied with the methodological and reporting guidelines in the field of acupuncture and moxibustion. Even though there were two studies regarding the methodological or reporting quality of NMAs in TCM, one English article excluded acupuncture and moxibustion [44] and one Chinese article included acupuncture and moxibustion literature incompletely [45]. Second, compared with published quality studies of NMAs in acupuncture and moxibustion, this review implemented a more comprehensive and detailed literature retrieval strategy. In addition, the unpublished studies and the references initially were also retrieved manually. As a result, the results were more credible.

This research also had presented some limitations: first, there was no specific methodological quality assessment tool for NMAs, even though AMSTAR2 was generally used in the quality evaluation of systematic review and meta-analysis. Second, in this study, the quality of each NMA was quantified by the assignment method, and there were some controversies on whether the weight of each item was consistent. Third, even if a comprehensive literature search strategy was used, there was no guarantee that all relevant literatures were identified. Finally, since only Chinese and English studies were included, there may be a lack of data to influence the results.

## 5. Conclusion

The NMAs methodological and report quality related to acupuncture and moxibustion were general, and there was still room for improvement in some aspects. For example, the researchers should design the scheme in advance before carrying out the study, design and carry out the study strictly in accordance with PICOS, and present the network structure, so as to improve the prospective and reliability of the study. Considering the importance of PRISMA-NMA checklist to NMA, we advise that the researchers should strictly follow the PRISMA-NMA checklist when writing a NMA.

## Figures and Tables

**Table 1:** Search strategy.

Source: PubMed; searched on: January 22, 2020	
Search	Query
#1	“acupuncture” [Ti/Ab] OR “moxibustion” [Ti/Ab]
#2	“network meta-analysis” [Ti/Ab] OR “mixed treatment meta-analysis” [Ti/Ab] OR “multiple treatment comparison meta-analysis” [Ti/Ab] OR “bayes meta-analysis” [Ti/Ab] OR “indirect comparison” [Ti/Ab]
#3	“network meta-analysis” [MeSH]
#4	“acupuncture” [MeSH] OR “moxibustion” [MeSH]
#5	#1 AND #2
#6	#3 AND #4
#7	#5 OR #6

**Table 2:** Basic characteristics of eligible studies.

Study ID	Disease	Sample size	Number of interventions	Description of interventions	Number of RCT	Outcome	Risk assessment tool
Fu et al. [15]	Acute gouty arthritis	4931	10	Western medicine, acupuncture + pricking blood and cupping, Chinese medicine + cupping, acupuncture and moxibustion, Chinese medicine, Western medicine + pricking blood and cupping, pricking blood and cupping, Western medicine + acupuncture, Western medicine + massage, and Western medicine + Chinese medicine	66	Effective rate	Jadad

Ding [16]	Abdominal distension after abdominal operation	2047	7	Acupoint application, acupoint injection, acupoint massage, moxibustion, TCM enema, TCM hot ironing, and routine nursing	22	Effective rate	Jadad

Li [17]	Poststroke depression	1895	13	Acupuncture + Chinese medicine, ear bean + Chinese medicine, electric acupuncture + Chinese medicine, moxibustion + Chinese medicine, auricular acupoint electroacupuncture + Chinese medicine, head acupuncture + acupoint embedding, auricular acupoint electroacupuncture + acupuncture, ear bean + acupuncture, acupuncture + acupoint injection, acupuncture + wuxing music, wuxing music + Chinese medicine, SSRIs, and acupuncture + moxibustion	23	Effective rate	Jadad

Bu et al. [18]	Optic atrophy	1369	11	Western medicine, acupuncture, acupuncture + moxibustion, ear point + Chinese medicine, electroacupuncture, acupuncture + Chinese and Western medicine, moxibustion + Chinese and Western medicine, electroacupuncture + Chinese and Western medicine, Chinese and Western medicine, Chinese medicine, and acupuncture + Western medicine	16	Effective rate, vision, horizon, and visual evoked potential	RoB

Zhang [19]	Ankylosing spondylitis	2208	9	Sulfasalazine, acupuncture, moxibustion, bee acupuncture, acupuncture + moxibustion, acupuncture + moxibustion + cupping, and moxibustion + bee needle	25	Effective rate	RoB

Song [20]	Polycystic ovary syndrome	4605	14	Acupuncture-medication therapy, Western medicine, acupuncture and moxibustion, acupuncture, acupuncture + ear points, moxibustion + Chinese medicine, acupuncture + ear points, acupuncture + placebo, placebo + Western medicine, Chinese medicine + Western medicine, placebo, acupoint thread-embedding therapy and medication, Chinese medicine, and moxibustion	39	Ovulation rate and pregnancy rate	RoB

Yang et al. [21]	Lumbar disc herniation	2589	4	Acupuncture, acupuncture + cupping, acupuncture + massage, and acupuncture + cupping + massage	30	Pain improvement, effectiveness, cure rate, recurrence rate, and economic indicators.	RoB

Li [22]	Primary dysmenorrhea	4600	6	Acupuncture, acupuncture + moxibustion, acupuncture + indirect moxibustion, warm acupuncture, electroacupuncture, and electroacupuncture + warm acupuncture	56	Effective rate, VAS, and dysmenorrhea symptom score	RoB

Jia et al. [23]	Chronic urticaria	1186	9	Acupoint catgut embedding, acupoint catgut embedding + loratadine, acupoint catgut embedding + mizolastine, acupoint catgut embedding + cetirizine, acupoint catgut embedding + epstein, loratadine, mizolastine, cetirizine, and epstein	15	Effective rate and adverse effect	RoB

Liu [24]	Knee osteoarthritis	3417	6	Hyaluronic acid (HA), needle knife, needle knife + HA, needle knife + internal medicine, needle knife + massage, and needle knife + acupuncture	29	Effective rate, VAS, and Lysholm function scores	Jadad

Feng [25]	Primary dysmenorrhea	10259	9	Multipoint + conventional Chinese medicine therapy, multipoint + Western medicine conventional therapy, single point + TCM, TCM, single point, multipoint, Western medicine routine therapy, and placebo	103	Effective rate, VAS, CMSS, and SF-MPQ	RoB

Liu [26]	Poststroke shoulder pain	1898	9	Rehabilitation, acupuncture, acupuncture + rehabilitation, acupuncture + massage, acupuncture + TCM fumigation + rehabilitation, acupuncture + massage + cupping, acupuncture + massage + rehabilitation, acupuncture + TCM + rehabilitation, and acupuncture + joint loosening + rehabilitation	25	Effective rate, FMA, and MBI	RoB

Yang et al. [27]	Premature ovarian insufficiency	3046	18	HRT, acupuncture, moxibustion, needle-warming moxibustion, electroacupuncture, catgut implantation at acupoint , TCM, acupuncture + TCM, moxibustion + TCM, electroacupuncture + TCM, acupoint application + TCM, auricular point sticking + TCM, acupressure + TCM, catgut implantation at acupoint + TCM, acupuncture + HRT, electroacupuncture + HRT, catgut implantation at acupoint + HRT, and acupuncture + TCM + HRT	43	Effective rate and adverse effect	RoB

Zhu et al. [28]	Chronic constipation	11032	10	Acupuncture, polyethylene glycol, lactulose, linaclotide, lubiprostone, bisacodyl, prucalopride, sham acupuncture, tegaserod, and placebo	40	Symptoms of chronic constipation and side effects	Jadad

Zheng [29]	Chronic functional constipation	4324	8	Acupuncture, mosapride, insoluble fiber, massage, mineral water, probiotic, TENS, and moxibustion	33	Weekly stool frequency, Bristol score, responder rate, and adverse event	RoB

Qin [30]	Chronic prostatitis/chronic pelvic pain syndrome	1203	7	Acupuncture, electroacupuncture, alpha-blockers, antibiotics, dual therapy, sham acupuncture, and placebo	12	NIH-CPSI QoL score	RoB

Li et al. [31]	Knee osteoarthritis	2065	7	Common manual acupuncture, electroacupuncture, fire needle, warm needle, placebo, sham needle, and education	16	WOMAC, stiffness, and physical function scores	RoB

Mo et al. [32]	Lumbar disc herniation	13075	4	Tuina, traction, acupuncture, and Chinese herbs	121	Invalid rate, cure rate, VAS, and JOA	RoB

Luo et al. [33]	Primary dysmenorrhea	1511	8	Traditional acupuncture, eye acupuncture, wrist-ankle acupuncture, superficial acupuncture, moxibustion, electroacupuncture, ear acupuncture, and abdominal acupuncture	17	Effective rate	RoB

Yeh et al. [34]	Psoriasis	869	6	Acupuncture, acupressure, acupoint bloodletting, acupoint catgut embedding, Chinese herbal medicine, and narrow-band ultraviolet B	10	PASI and TCM	Jadad RoB

Chen et al. [35]	Migraine	3656	9	Acupuncture, flunarizine, metoprolol, propranolol, propranolol + flunarizine, sham acupuncture, topiramate, usual care, and waiting list	19	Migraine episodes, the number of migraine days, migraine frequency, responder rate, and adverse event rate	RoB

Tan et al. [36]	Essential hypertension	2649	15	Electroacupuncture, moxibustion, warm needle therapy, sham acupuncture, behavioral therapy, angiotensin-converting enzyme inhibitors (ACEIs), angiotensin receptor blockers (ARBs), calcium channel blocker (CCB), beta-blocker, acupuncture-combined ACEI, acupuncture-combined CCB, acupuncture-combined behavior, electroacupuncture-combined CCB, sham acupuncture, and nontreatment	31	posttreatment BP changes, response rate, and MACE	RoB

Li et al. [37]	Myofascial pain syndrome	1692	22	Placebo-sham, MA, EA, DN, MET, TCT, MT, LTrP-I, MDIMST, MSN, TTM, BTX-A-TrP-I, FN, SWAM, EA&ESNC, SPM, DN&MET, BTrP-I, TrP-DN&EDU, Stretch, DN&Stretch, laser, and PT	33	Pain measurement (VAS, NRS, and PPT), adverse events (ROM), and functional status	RoB

Zhu et al. [38]	Diarrhea-predominant irritable bowel syndrome	9369	7	Acupuncture, sham acupuncture, pinaverium bromide, alosetron, eluxadoline, ramosetron, and rifaximin	29	Effective rate, side effects (constipation and rash), and common acupuncture points.	Jadad

Xiong and Chen [39]	Diabetic peripheral neuropathy	2602	7	Manual acupuncture, electroacupuncture, needle knocking acupuncture, warm needling and moxibustion, mecobalamin, no interventions, and vitamin B	40	global symptom improvement	non

Yang et al. [40]	Heart failure	2116	5	Acupuncture, moxibustion, acupoint application, acupoint injection, and warming acupuncture-moxibustion	26	HFC and LVEF	RoB

Zhang et al. [41]	Obesity	2283	6	AAS, EA, ACE, WA, AR: acupuncture and related therapies and combination of acupuncture and related therapies.	34	BW, BMI, and adverse events	RoB

Corbett 2013 [42]	Knee osteoarthritis	9709	8	Interferential therapy, acupuncture, TENS, pulsed electrical stimulation, balneotherapy, aerobic exercise, sham acupuncture, and muscle-strengthening exercise	114	WOMAC pain	RoB

Wang et al. [43]	Chronic fatigue syndrome	2255	5	CbAM, SAM, Chinese herbal medicine, Western medicine, and sham acupuncture	31	Effective rate	RoB

Note: CMSS, the Cox Menstrual Symptom Scale; FMA, Fugl－Meyer motor assessment scale; MBI, modified Barthel index; TCM, traditional Chinese medicine; MACE, major adverse cardiovascular events; HFC, changes in heart function classification; LVEF, left ventricular ejection fraction; AAS, auricular acupoint stimulation; WA, warming acupuncture; ACE, acupoint catgut embedding; EA, electroacupuncture; AR, acupuncture and related therapies.

**Table 3:** Methodological quality assessment of the included NMAs.

Item	1	2	3	4	5	6	7	8	9	10	11	12	13	14	15	16	Score
Fu et al. [15]	Y	N	N	P	Y	Y	N	P	P	N	N	N	N	N	Y	N	5.5
Ding [16]	Y	N	N	P	Y	N	N	Y	Y	N	N	N	N	N	N	P	5
Li [17]	Y	N	N	P	Y	N	N	P	Y	N	Y	N	P	N	N	N	5.5
Bu et al. [18]	Y	N	N	Y	Y	Y	N	P	Y	N	P	N	P	N	N	P	7
Zhang [19]	Y	N	N	Y	N	Y	N	P	Y	N	Y	N	P	Y	N	P	6
Song [20]	Y	N	N	Y	Y	Y	N	Y	Y	N	P	P	P	Y	Y	P	8
Yang et al. [21]	Y	N	N	P	N	Y	N	Y	Y	N	Y	N	Y	Y	N	P	8
Li [22]	Y	N	N	Y	Y	Y	N	Y	Y	N	Y	Y	Y	Y	P	P	11
Jia et al. [23]	Y	N	N	P	Y	N	N	Y	Y	N	Y	Y	Y	Y	P	P	9.5
Liu [24]	Y	N	N	Y	Y	Y	N	Y	Y	N	Y	Y	N	Y	N	P	9.5
Feng [25]	Y	N	N	Y	P	Y	N	Y	Y	N	Y	Y	Y	Y	P	N	10
Liu [26]	Y	N	N	P	N	N	N	Y	Y	N	Y	N	P	N	N	P	5.5
Yang et al. [27]	Y	N	N	Y	Y	Y	N	Y	Y	N	Y	Y	Y	Y	Y	P	11
Zhu et al. [28]	N	N	N	Y	Y	Y	N	N	Y	N	Y	N	N	N	Y	P	6.5
Zheng [29]	P	Y	Y	Y	Y	Y	N	P	Y	N	Y	N	Y	Y	P	Y	11
Qin [30]	Y	N	N	Y	Y	Y	N	Y	Y	N	Y	Y	Y	Y	N	Y	11
Li et al. [31]	Y	N	N	P	Y	Y	N	P	Y	N	Y	Y	Y	Y	Y	Y	11
Mo et al. [32]	P	N	N	Y	Y	Y	N	P	Y	N	P	N	N	N	Y	Y	7.5
Luo et al. [33]	Y	N	N	P	N	N	N	P	Y	N	Y	Y	N	N	Y	P	6.5
Yeh et al. [34]	P	N	N	P	Y	Y	N	P	Y	N	Y	Y	Y	Y	Y	P	10
Chen et al. [35]	P	Y	N	Y	Y	N	N	P	Y	N	Y	N	N	P	N	Y	7.5
Tan et al. [36]	Y	N	N	P	Y	Y	N	Y	Y	N	Y	Y	Y	N	Y	Y	10.5
Li et al. [37]	Y	Y	N	Y	Y	Y	N	Y	Y	N	Y	Y	Y	N	N	P	10.5
Zhu et al. [38]	P	N	N	Y	Y	Y	N	P	Y	N	Y	Y	Y	P	Y	P	10
Xiong and Chen [39]	P	N	N	P	N	N	N	P	N	N	N	N	N	N	N	Y	2.5
Yang et al. [40]	Y	Y	N	P	Y	Y	N	P	Y	N	Y	Y	Y	N	Y	Y	11
Zhang et al. [41]	Y	N	N	P	Y	Y	N	Y	Y	N	Y	Y	Y	N	N	Y	9.5
Corbett 2013 [42]	Y	Y	Y	Y	Y	Y	N	P	Y	N	Y	Y	Y	Y	Y	Y	13.5
Wang et al. [43]	Y	N	N	P	N	Y	N	Y	Y	N	Y	Y	Y	Y	Y	Y	10.5
Score	20	5	2	15	22	22	0	14	27	0	23	16	16	14	13	11	8.64/9.5

Y, yes (1 point); N, no (0 point); P, partial satisfaction (0.5 point). Item 1, did the research questions and inclusion criteria for the review include the components of PICO? Item 2, did the report of the review contain an explicit statement that the review methods were established prior to the conduct of the review and did the report justify any significant deviations from the protocol? Item 3, did the review authors explain their selection of the study designs for inclusion in the review? Item 4, did the review authors use a comprehensive literature search strategy? Item 5, did the review authors perform study selection in duplicate? Item 6, did the review authors perform data extraction in duplicate? Item 7, did the review authors provide a list of excluded studies and justify the exclusions? Item 8, did the review authors describe the included studies in adequate detail? Item 9, did the review authors use a satisfactory technique for assessing the risk of bias (RoB) in individual studies that were included in the review? Item 10, did the review authors report on the sources of funding for the studies included in the review? Item 11, if meta-analysis was performed, did the review authors use appropriate methods for statistical combination of results? Item 12, if meta-analysis was performed, did the review authors assess the potential impact of RoB in individual studies on the results of the meta-analysis or other evidence synthesis? Item 13, did the review authors account for RoB in individual studies when interpreting/ discussing the results of the review? Item 14, did the review authors provide a satisfactory explanation for, and discussion of, any heterogeneity observed in the results of the review? Item 15, if they performed quantitative synthesis, did the review authors carry out an adequate investigation of publication bias (small study bias) and discuss its likely impact on the results of the review? Item 16, did the review authors report any potential sources of conflict of interest, including any funding they received for conducting the review?

**Table 4:** Summary of methodological quality assessment.

Number	Item	Completely reported		Partially reported		Not reported	
		Frequency (%)	95% CI	Frequency (%)	95% CI	Frequency (%)	95% CI
1	Components of PICO question?	22 (75.86%)	(0.58, 0.94)	6 (20.69%)	(−0.12, 0.53)	1 (3.45%)	(−0.32, 0.39)
2	Review protocol?	5 (17.24%)	(−0.16, 0.50)	0		24 (82.75%)	(0.68, 0.98)
3	Explanation of study design?	2 (6.90%)	(−0.28, 0.42)	0		27 (93.10%)	(0.84, 1.03)
4	Comprehensive literature search strategy	16 (55.17%)	(0.31, 0.80)	13 (44.83%)	(0.18, 0.72)	0	
5	Study selection in duplicate?	22 (75.86%)	(0.58, 0.94)	1 (3.45%)	(−0.32, 0.39)	6 (20.69%)	(−0.12, 0.53)
6	Data extraction in duplicate?	22 (75.86%)	(0.58, 0.94)	0		7 (24.14%)	(−0.08, 0.56)
7	List of excluded studies and justify the exclusions?	0		0		29 (100%)	
8	Study characteristics	14 (48.27%)	(0.22, 0.74)	14 (48.27%)	(0.22, 0.74)	1 (3.45%)	(−0.32, 0.39)
9	Satisfactory technique for assessing risk of bias?	27 (93.10%)	(0.84, 1.03)	1 (3.45%)	(−0.32, 0.39)	1 (3.45%)	(−0.32, 0.39)
10	Sources of funding for each study?	0		0		29 (100%)	
11	Appropriate methods?	23 (79.31%)	(0.63, 0.96)	3 (10.34%)	(−0.24, 0.45)	3 (10.34%)	(−0.24, 0.45)
12	Assess potential impact of risk of bias on the results?	16 (55.17%)	(0.31, 0.80)	1 (3.45%)	(−0.32, 0.39)	12 (41.38%)	(0.14, 0.69)
13	Account for risk of bias when interpreting/discussing?	16 (55.17%)	(0.31, 0.80)	5 (17.24%)	(−0.16, 0.50)	8 (27.59%)	(−0.03, 0.59)
14	Satisfactory explanation for and discussion of any heterogeneity?	14 (48.27%)	(0.29, 0.81)	2 (6.90%)	(−0.28, 0.42)	13 (44.83%)	(0.18, 0.72)
15	Publication bias (small sample bias) assessed and discussed?	13 (44.83%)	(0.18, 0.72)	4 (13.79%)	(−0.20, 0.48)	12 (41.38%)	(0.14, 0.69)
16	Publication bias (small sample bias) assessed and discussed?	11 (37.93%)	(0.09, 0.67)	15 (51.72%)	(0.26, 0.77)	3 (10.34%)	(−0.24, 0.45)

**Table 5:** Reporting quality assessment of the included NMAs.

Item	Section/topic	Fu 2019	Ding 2019	Li 2018	Bu 2017	Zhang 2018	Song 2019	Yang 2016	Li 2017	Jia 2018	Liu 2016	Feng 2018	Liu 2019	Yang 2017	Zhu 2018	Zheng 2018
1	Title	1	1	1	1	1	1	1	1	1	1	1	1	1	1	1
2	Structured summary	0.5	0.5	0.5	0.5	0.5	0.5	0.5	0.5	0.5	0.5	0.5	0.5	0.5	0.5	1
3	Rationale	1	0.5	1	0.5	1	1	1	1	1	1	0.5	1	1	1	1
4	Objectives	1	1	1	0.5	1	1	1	1	1	1	1	1	1	1	1
5	Protocol and registration	0	0	0	0	0	0	0	0	0	0	0	0	0	0	1
6	Eligibility criteria	1	1	0.5	0.5	0.5	1	1	1	1	1	1	1	1	1	0.5
7	Information sources	1	1	1	1	1	1	0.5	1	1	1	1	1	1	1	1
8	Search	0	0	0	0	0	0	0	1	0	1	1	0	1	0	1
9	Study selection	0.5	1	1	1	0	1	0	1	1	1	1	0	1	1	1
10	Data collection process	0.5	0.5	0.5	1	0.5	0.5	0.5	1	0.5	0.5	1	0.5	0.5	0.5	0.5
11	Data items	0.5	0.5	1	0.5	0.5	0.5	0.5	0.5	0.5	0.5	0.5	0.5	0.5	0.5	0.5
S1	Geometry of the network	1	0	1	0	1	1	0	1	1	1	1	1	1	1	1
12	Risk of bias within individual studies	1	1	1	1	1	1	1	1	1	1	1	1	1	1	1
13	Summary measures	1	0.5	1	0.5	0.5	0.5	0.5	1	1	1	1	1	1	1	1
14	Planned methods of analysis	1	0	1	1	0	1	1	1	1	1	1	1	1	1	1
S2	Assessment of inconsistency	1	0	1	1	1	1	1	1	1	0	1	1	1	1	1
15	Risk of bias across studies	1	0	0	1	0	1	0	1	1	1	1	0	1	1	1
16	Additional analyses	0	0	0	0	0	1	0	0	1	1	1	0	0	1	1
17	Study selection	1	1	0.5	1	0.5	0.5	1	1	1	1	1	1	1	1	1
S3	Presentation of network structure	1	1	1	1	1	1	0	1	1	1	1	1	1	1	1
S4	Summary of network geometry	1	0.5	0.5	0.5	0.5	0.5	0	0.5	0.5	0.5	0.5	0.5	0.5	0	0.5
18	Study characteristics	1	1	1	1	1	1	1	1	1	1	1	1	1	1	1
19	Risk of bias within studies	1	1	1	1	1	1	1	1	1	1	1	1	1	1	0
20	Results of individual studies	0	0	0	0	0	0	0	1	1	0	1	0	1	1	0
21	Synthesis of results	1	1	1	1	1	0.5	1	1	1	1	1	1	1	1	1
S5	Exploration for inconsistency	1	0	0	1	1	1	1	1	1	1	1	1	0	0	0
22	Risk of bias across studies	1	0	0	0	0	1	0	1	1	0	1	0	0	1	0
23	Results of additional analyses	0	0	0	0	1	0	0	0	0	1	0	0	0	1	0
24	Summary of evidence	1	0	1	0	0.5	1	1	1	1	1	1	1	1	1	1
25	Limitations	1	1	1	1	1	1	1	1	1	1	1	1	1	1	1
26	Conclusions	1	1	0.5	1	1	0.5	1	1	1	1	1	1	1	1	1
27	Funding	0	1	0	1	1	1	1	1	0	1	0	1	1	0	1
	Summary	24	17	20	20.5	20	24	18.5	27.5	26	26	27	22	25	25.5	25

Item		Qin 2016	Li 2018	Mo 2019	Luo 2018	Yeh 2016	Chen 2019	Tan 2019	Li 2017	Zhu 2018	Xiong 2018	Yang 2019	Zhang 2018	S. 2013	Wang 2017	

1		1	1	1	1	1	1	1	1	1	1	1	1	1	1	
2		0.5	0.5	0.5	0.5	0.5	1	0.5	1	0.5	0.5	1	0.5	1	0.5	
3		1	1	1	1	1	1	1	1	1	1	1	1	1	1	
4		1	1	1	1	1	1	1	1	1	1	1	1	1	1	
5		0	0	0	0	0	1	0	1	0	0	1	0	1	0	
6		1	1	0.5	1	0.5	0.5	1	1	0.5	0.5	1	1	1	0.5	
7		1	0.5	1	1	0.5	1	1	1	1	0.5	0.5	1	1	1	
8		1	0	1	0	0	0	1	1	1	0	1	1	0	0	
9		1	1	1	0.5	1	1	1	1	1	0	1	1	1	0	
10		1	0.5	0.5	0.5	0.5	0.5	0.5	0.5	0.5	0	0.5	1	0.5	0.5	
11		1	0.5	0.5	0.5	0.5	0.5	1	0.5	0.5	0.5	0.5	0.5	0.5	0.5	
S1		1	1	0	0	0	0	0	1	1	0	0	0	0	0	
12		1	1	1	1	1	1	1	1	1	0	1	1	1	1	
13		1	1	1	1	0.5	0.5	0.5	1	0.5	0	1	1	0.5	0.5	
14		1	1	0	1	1	1	0	1	1	0.5	1	1	1	0	
S2		1	1	1	0	0	1	1	1	1	1	1	0	1	0	
15		1	1	1	1	1	1	1	1	1	0	1	0	1	1	
16		1	1	0	0	0	0	0	1	1	0	1	1	1	1	
17		1	1	1	1	1	0.5	1	1	1	1	1	1	1	1	
S3		1	1	1	1	1	1	1	1	1	1	1	1	1	1	
S4		0.5	0.5	0.5	0.5	0.5	0.5	0.5	0.5	0.5	0.5	0.5	0.5	0.5	0.5	
18		1	1	1	1	1	1	1	1	1	0.5	1	1	0.5	1	
19		1	1	1	1	1	1	1	1	1	0	1	1	0.5	1	
20		1	1	1	1	1	1	1	1	1	0	1	1	0	1	
21		1	1	1	1	1	1	1	1	1	1	1	1	0.5	1	
S5		1	1	1	0	1	1	1	1	1	1	1	1	0	0	
22		0	1	1	1	0	1	1	0	1	0	1	0	0	0	
23		1	1	0	0	0	0	0	0	1	0	0	0	1	1	
24		1	1	1	1	1	1	1	1	1	0	1	1	1	1	
25		1	1	1	1	1	1	1	1	1	0	1	1	1	1	
26		1	1	1	1	1	1	1	1	1	1	1	1	1	1	
27		1	1	1	0	0	1	1	1	0	1	1	1	1	1	
Score		29	27.5	24.5	21.5	20.5	25	25	28.5	27	13.5	28	24.5	23.5	19	24.5/23.62

**Table 6:** Summary of reporting quality assessment.

Section	Item		Completely reported		Partially reported		Not reported	
			Frequency (%)	95% CI	Frequency (%)	95% CI	Frequency (%)	95% CI
Title	1	Title	29 (100%)		0		0	
Abstract	2	Structured summary	5 (17.24%)	(−0.16, 0.50)	24 (82.76%)	(0.68, 0.98)	0	

Introduction	3	Rationale	26 (89.65%)	(0.78, 1.01)	3 (10.34%)	(−0.24, 0.45)	0	
	4	Objectives	28 (96.55%)	(0.90, 1.03)	1 (3.45%)	(−0.32, 0.39)	0	

Methods	5	Protocol and registration	5 (17.24%)	(−0.16, 0.50)	0		24 (82.76%)	(0.68, 0.98)
	6	Eligibility criteria	19 (65.52%)	(0.44, 0.87)	10 (34.48%)	(0.05, 0.64)	0	
	7	Information sources	22 (75.86%)	(0.58, 0.94)	5 (17.24%)	(−0.16, 0.50)	0	
	8	Search	12 (41.38%)	(0.14, 0.69)	0		17 (58.62%)	(0.35, 0.82)
	9	Study selection	22 (75.86%)	(0.58, 0.94)	2 (6.70%)	(−0.28, 0.42)	5 (17.24%)	(−0.16, 0.50)
	10	Data collection process	5 (17.24%)	(−0.16, 0.50)	23 (79.31%)	(0.63, 0.96)	1 (3.45%)	(−0.32, 0.39)
	11	Data items	3 (10.34%)	(−0.24, 0.45)	26 (89.65%)	(0.78, 1.01)	0	
	S1	Geometry of the network	16 (55.17%)	(0.31, 0.80)	0		13 (44.83%)	(0.18, 0.72)
	12	Risk of bias within individual studies	28 (96.55%)	(0.90, 1.03)	0		1 (3.45%)	(−0.32, 0.39)
	13	Summary measures	18 (62.07%)	(0.40, 0.85)	11 (37.93%)	(0.09, 0.67)	0	
	14	Planned methods of analysis	23 (79.31%)	(0.63, 0.96)	1 (3.45%)	(−0.32, 0.39)	5 (17.24%)	(−0.16, 0.50)
	S2	Assessment of inconsistency	23 (79.31%)	(0.63, 0.96)	0		6 (20.69%)	(−0.12, 0.53)
	15	Risk of bias across studies	22 (75.86%)	(0.58, 0.94)	0		7 (24.14%)	(−0.08, 0.56)
	16	Additional analyses	14 (48.27%)	(0.29, 0.81)	0		15 (51.72%)	(0.26, 0.77)

Results	17	Study selection	25 (86.21%)	(0.73, 0.99)	4 (13.79%)	(−0.20, 0.48)	0	
	S3	Presentation of network structure	18 (62.07%)	(0.40, 0.85)	0		1 (3.45%)	(−0.32, 0.39)
	S4	Summary of network geometry	1 (3.45%)	(−0.32, 0.39)	26 (89.65%)	(0.78, 1.01)	2 (6.70%)	(−0.28, 0.42)
	18	Study characteristics	27 (93.10%)	(0.84, 1.03)	2 (6.70%)	(−0.28, 0.42)	0	
	19	Risk of bias within studies	26 (89.65%)	(0.78, 1.01)	1 (3.45%)	(−0.32, 0.39)	2 (6.70%)	(−0.28, 0.42)
	20	Results of individual studies	17 (58.62%)	(0.35, 0.82)	0		12 (41.38%)	(0.14, 0.69)
	21	Synthesis of results	27 (93.10%)	(0.84, 1.03)	2 (6.70%)	(−0.28, 0.42)	0	
	S5	Exploration for inconsistency	21 (72.41%)	(0.53, 0.92)	0		8 (27.59%)	(−0.03, 0.59)
	22	Risk of bias across studies	13 (44.83%)	(0.18, 0.72)	0		16 (55.17%)	(0.31, 0.80)
	23	Results of additional analyses	8 (27.59%)	(−0.03, 0.59)	0		21 (72.41%)	(0.53, 0.92)

Discussion	24	Summary of evidence	25 (86.21%)	(0.73, 0.99)	1 (3.45%)	(−0.32, 0.39)	3 (10.34%)	(−0.24, 0.45)
	25	Limitations	28 (96.55%)	(0.90, 1.03)	0		1 (3.45%)	(−0.32, 0.39)
	26	Conclusions	27 (93.10%)	(0.84, 1.03)	2 (6.70%)	(−0.28, 0.42)	0	
	27	Funding	21 (72.41%)	(0.53, 0.92)	0		8 (27.59%)	(−0.03, 0.59)

## Data Availability

The data used to support the findings of this study are available from the corresponding author upon request.

## Abbreviations

AMSTAR2: A measurement tool to assess the methodological quality of systematic reviews
PRISMA-NMA: PRISMA extension statement for reporting of systematic reviews incorporating network meta-analysis of health care interventions
NMA: Network meta-analysis.
